# Multifactorial chromosomal variants regulate polymyxin resistance in extensively drug-resistant *Klebsiella pneumoniae*

**DOI:** 10.1099/mgen.0.000158

**Published:** 2018-02-12

**Authors:** Miranda E. Pitt, Alysha G. Elliott, Minh Duc Cao, Devika Ganesamoorthy, Ilias Karaiskos, Helen Giamarellou, Cely S. Abboud, Mark A. T. Blaskovich, Matthew A. Cooper, Lachlan J. M. Coin

**Affiliations:** ^1^​ Institute for Molecular Bioscience, The University of Queensland, Brisbane, Australia; ^2^​ 6th Department of Internal Medicine, Hygeia General Hospital, Athens, Greece; ^3^​ Instituto Dante Pazzanese de Cardiologia, São Paulo, Brazil

**Keywords:** *Klebsiella pneumoniae*, antibiotic resistance, polymyxin, chromosomal variants

## Abstract

Extensively drug-resistant *Klebsiella pneumoniae* (XDR-KP) infections cause high mortality and are disseminating globally. Identifying the genetic basis underpinning resistance allows for rapid diagnosis and treatment. XDR isolates sourced from Greece and Brazil, including 19 polymyxin-resistant and five polymyxin-susceptible strains, were subjected to whole genome sequencing. Seventeen of the 19 polymyxin-resistant isolates harboured variations upstream or within *mgrB*. The most common mutation identified was an insertion at nucleotide position 75 in *mgrB* via an IS*Kpn26*-like element in the ST258 lineage and IS*Kpn13* in one ST11 isolate. Three strains acquired an IS*1* element upstream of *mgrB* and another strain had an IS*Kpn25* insertion at 133 bp. Other isolates had truncations (C28STOP, Q30STOP) or a missense mutation (D29E) affecting *mgrB*. Complementation assays revealed all *mgrB* perturbations contributed to resistance. Missense mutations in *phoQ* (T281M, G385C) were also found to facilitate resistance. Several variants in *phoPQ* co-segregating with the IS*Kpn26*-like insertion were identified as potential partial suppressor mutations. Three ST258 samples were found to contain subpopulations with different resistance-conferring mutations, including the IS*Kpn26*-like insertion colonizing with a novel mutation in *pmrB* (P158R), both confirmed via complementation assays. These findings highlight the broad spectrum of chromosomal modifications which can facilitate and regulate resistance against polymyxins in *K. pneumoniae*.

## Data Summary

Whole genome sequencing of the 24 clinical isolates has been deposited under BioProject PRJNA307517 (www.ncbi.nlm.nih.gov/bioproject/PRJNA307517).

**Impact Statement**

*Klebsiella pneumoniae* contributes to a high abundance of nosocomial infections and the rapid emergence of antimicrobial resistance hinders treatment. Polymyxins are predominantly utilized to treat multidrug-resistant infections, but resistance to the polymyxins is arising. This increasing prevalence in polymyxin resistance is particularly evident in Greece and Brazil. Identifying the genomic variations conferring resistance in clinical isolates from these regions will help with potentially detecting novel variants and tracing the spread of particular strains. This study commonly found mutations in the gene *mgrB*, the negative regulator of PhoPQ, known to cause resistance in *K. pneumoniae*. In the remaining isolates, missense mutations in *phoQ* were accountable for resistance. Multiple novel mutations were detected to be segregating with *mgrB* perturbations. This was either due to a mixed heterogeneous sample of two polymyxin-resistant strains, or because of multiple mutations within the same strain. Of interest was the validation of novel mutations in *phoPQ* segregating with a previously known IS*Kpn26*-like element in disrupted *mgrB* isolates. Complementation of these *phoPQ* mutations revealed a reduction in minimum inhibitory concentrations and suggests the first evidence of partial suppressor mutations in *K. pneumoniae*. This research builds upon our current understanding of heterogeneity, lineage-specific mutations and regulatory variations relating to polymyxin resistance.

## Introduction

*Klebsiella pneumoniae* (KP) strains classified as extensively drug-resistant (XDR) are rapidly emerging due to the dissemination of plasmid-encoded resistance towards aminoglycosides, β-lactams, fluoroquinolones and carbapenems [1]. Notably, carbapenem-resistant KP have been linked to high morbidity and an overall mortality of 48 % in infected patients [2]. Polymyxin B and colistin (polymyxin E) are now one of the last viable therapeutic options [3]. Unfortunately, resistance to this last-line antibiotic class is an increasing global burden, with countries particularly impacted including Asia (South Korea [4, 5], India [6, 7]), Europe (Greece [8–10]), Italy [10, 11]) and Latin America (Brazil [12, 13]). Mortality is influenced by polymyxin resistance typically occurring on a multidrug-resistant (MDR) or XDR background; nephrotoxicity leads to suboptimal dosing as well as inadequacies in detection of heteroresistance [10, 14]. As a result, there is considerable uncertainty regarding the mortality associated with polymyxin-resistant infections. Combining several clinical cohorts has provided overall mortality estimates ranging from 20 to 100 % [10].

Polymyxins infiltrate Gram-negative bacteria via initial binding to the basal component of lipopolysaccharide, lipid A. This causes the displacement of Mg^2+^ and Ca^2+^, disrupting the outer and inner membrane integrity, resulting in leakage of cytoplasmic contents and subsequent cell death, but the exact mechanism involved remains elusive [15, 16]. Inhibition of an intracellular target, the type II NADH-quinolone oxidoreductases, embedded in the inner membrane has also been identified [17]. An exposure in KP leads to the emergence of polymyxin resistance by triggering the activation of the two-component regulatory systems, PmrAB and PhoPQ [18–20]. These systems regulate a pathway that modulates *pmrC* and the *pmrHFIJKLM* operon, facilitating the addition of phosphoethanolamine (pEtN) and/or 4-amino-4-deoxy-l-arabinose to lipid A phosphate groups, resulting in impaired polymyxin binding interactions [21–23]. Disruption of *mgrB*, the negative regulator of PhoPQ, has been commonly observed in isolates of clinical origin [8, 24]. The constitutive up-regulation of *pmrC* and the *pmrHFIJKLM* operon incurs a minimal fitness cost and appears to be stable, with few reports of reversions [25, 26]. Heteroresistant populations, in which only a subset of bacteria are resistant in a phenotypically susceptible sample, have been reported in KP, which complicates diagnosis [27]. The emergence of pandrug-resistant KP is of grave concern [28] and this acquisition of resistance is further exacerbated by the recently reported plasmid-encoded colistin resistance gene *mcr-1*, which encodes a pEtN transferase enzyme [29, 30]. The presence of *mcr* genes in KP is currently a rarity with only a few reports of *mcr-1*, -*1.2* and -*3* [31–33]. Göttig *et al.* recently established a fitness cost associated with KP harbouring *mcr-1*, in contrast to *Escherichia coli*, which may explain the lack of isolates acquiring this gene [34]. This study aimed to investigate XDR-KP clinical isolates arising in Greece and Brazil during 2012–2014 to identify and validate genetic variants contributing to resistance. These variants were compared to prior clinical isolates to ascertain if these mutations have been previously detected globally.

## Methods

### Bacterial isolates

Polymyxin-resistant XDR-KP clinical isolates were acquired from the Hygeia General Hospital, Athens, Greece, and Instituto Dante Pazzanese de Cardiologia, Brazil, from patients in 2012–2014. These isolates were sampled at random (non-sequential). We also obtained five polymyxin-susceptible strains which were utilized as a genomic background reference and a negative control for complementation assays. Cultures were supplied as stabs/slants or on agar, and were subsequently cultured in nutrient broth. Cultures were made to 20 % (v/v) glycerol and stored at −80 °C. When required for assay or extraction, glycerol stocks were struck out to obtain single colonies on either nutrient agar or tryptic soy agar with 5 % defibrinated sheep blood. Reference strains included *E. coli* (ATCC 25922) and *Klebsiella* spp. (ATCC 13883, ATCC 700603, ATCC BAA-2146), which were obtained from the American Type Culture Collection (ATCC).

### Antimicrobial susceptibility assays

Species identification and susceptibility profiles of clinical isolates from Greece and Brazil were evaluated in the clinic using the VITEK2 system (bioMérieux). Strains were further validated at the Institute for Molecular Bioscience (IMB) (The University of Queensland, Australia) using the standard Clinical and Laboratory Standards Institute (CLSI) approved broth microdilution (BMD) methods with cation-adjusted Mueller-Hinton broth (caMHB). Resistance was determined as per CLSI guidelines [35] except for tigecycline and fosfomycin for which The European Committee on Antimicrobial Susceptibility Testing (EUCAST) (Version 7.1, 2017) (see http://www.eucast.org) guidelines were implemented. Classification of isolates as MDR or XDR was determined through guidelines previously outlined [36].

### DNA extraction

Isolates selected for sequencing exhibited a polymyxin-resistant XDR profile with five polymyxin-susceptible strains included to discern the mutations in *mgrB*, *pmrAB* and *phoPQ* segregating with susceptible isolates. DNA was extracted from overnight cultures using the DNeasy Blood and Tissue Kit (Qiagen) with the additional enzymatic lysis buffer pre-treatment as per the manufacturer’s instructions. DNA was quantified with Qubit3.0 (ThermoFisher Scientific).

### DNA library preparation and sequencing

Library preparation was performed using the Nextera XT kit (Illumina) with 1 ng input of DNA as per the manufacturer’s instructions. Quality of libraries was checked using a 2100 Bioanalyzer (Agilent Technologies). Libraries were prepared using the 2×300 v3 MiSeq kit and sequenced on an Illumina MiSeq with 300 bp paired-end sequencing reads and >100× coverage per sample.

### Sequencing analysis

Paired-end reads were trimmed with Trimmomatic [37] and assembled using SPAdes [38]. The Rapid Annotation using Subsystem Technology (RAST) was utilized to annotate assembled genomes [39]. Assemblies were also uploaded to the Centre for Genomic Epidemiology (CGE) to identify sequence types (STs) (MultiLocus Sequence Typing Server 1.8 [40]) and acquired antibiotic resistance genes (ResFinder 3.0 [41]). A neighbour-joining tree was reconstructed using the 2358 *Klebsiella pneumoniae*/*quasipneumoniae*/*variicola* genes known to form the core genome multi-locus sequence type (cgMLST) using Ridom SeqSphere +v4.0.1 software [42]. Complete assemblies of publicly available reference genomes were obtained from www.ncbi.nlm.nih.gov/gen ome/?term=klebsiella. ST references included HS11286 (ST11), MS6671 (ST147), and NJST258_1 and NJST258_2 (ST258). Species references were *K. quasipneumoniae* (ATCC 700603, HKUOPA4) and *K. variicola* (At-22, GJ1).

### Variant detection

Variants both in and flanking the genes *pmrAB*, *phoPQ* and *mgrB* were examined and sequence reads of all strains were aligned to the assembly of 20_GR_12, a polymyxin-susceptible ST258 strain with the least number of contigs, using BWA-MEM [43]. The alignment was analysed through FreeBayes [44] to identify single nucleotide and small indel variation, using a diploid analysis. The diploid analysis displays reads mapping to the predominant variant in the isolate and if a variant in lower abundance (≥20 % of reads) was identified, this was classified as heterogeneity. The effects of variations were determined by snpEff [45]. The impact on protein sequence was further confirmed by the Protein Variation Effect Analyzer (PROVEAN) [46]. For the analysis of large chromosome changes, the gene sequences including 300 bp flanking were extracted from the assemblies. A multiple alignment of each gene was reconstructed from the pairwise alignment to the longest gene sequence. Furthermore, assemblies of the five genes were aligned to the reference polymyxin-susceptible isolate ATCC 700603 to discern lineage- and species-specific variation.

### Insertion sequence element validation

ISFinder [47] was used for the identification of insertion sequence (IS) elements. To confirm disruptive IS elements, *mgrB* was amplified with the primers displayed in Table S1 (available in the online version of this article) via 2× Phusion HF master mix (Invitrogen) under the following cycling conditions: 98 °C for 10 s, 50 °C for 30 s and 72 °C for 60 s (35×). Amplicon identity was validated via Sanger sequencing.

### Complementation assays

The contribution of variants to resistance was validated through complementation assays as previously described [48]. Briefly, genes (Table S1) were amplified from a polymyxin-susceptible isolate, 20_GR_12, and cloned into the pCR-Blunt II-TOPO vector via the Zero Blunt TOPO PCR cloning kit (Invitrogen). Chemically competent *E. coli* TOP10 cells were transformed and selected by the addition of 50 mg l^−1^ kanamycin in Mueller-Hinton agar (MHA). Isolation of plasmids was done via the PureLink Quick Plasmid Miniprep Kit (Invitrogen). The methodology for preparing electrocompetent cells and complementation assays was kindly provided by Dr Aurélie Jayol and Professor Patrice Nordmann. Briefly, overnight cultures were subcultured into 200 ml Luria-Bertani broth (1 : 100 dilution) and grown to an OD_600_ of 0.6±0.05. Cells were chilled on ice before centrifugation (10 000 r.p.m., 10 min, 4 °C), washed twice with ice cold 10 % glycerol and concentrated to 500 µl. KP isolates were transformed via electroporation (25 µF, 200 Ω, 1.25 kV cm^−1^) with a Gene Pulser (Bio-Rad Laboratories). Selection was accomplished through supplementation of ≥500 mg l^−1^ zeocin on MHA plates. Transformed colonies (*n*≥2) were acquired and placed in MHB containing 1500 mg l^−1^ zeocin and 1 mM isopropyl *β-*d-1-thiogalactopyranoside (Sigma Aldrich). If polymyxin susceptibility was not restored upon complementation, genes harbouring mutations were further amplified and introduced into 20_GR_12. To discern the impact of additional mutations in *phoPQ* and *pmrB* segregating with disrupted *mgrB*, mutant genes were introduced into a polymyxin-resistant isolate only harbouring an IS element *mgrB* disruption, 7_GR_13. Controls included transformation of wild-type (WT) genes into 20_GR_12, sequencing of amplicons prior to introduction in the vector and in KP-transformed strains undergoing a plasmid extraction, and further PCR of the multiple cloning site. Antimicrobial testing against colistin and polymyxin B were conducted as described above.

## Results

### Characterization of clinical isolates

KP isolates were all characterized in the hospital microbiology facility using VITEK2 cards. Several discrepancies were detected between VITEK2 and broth microdilution (BMD) results (Tables 1 and S2), predominantly the level of resistance towards aminoglycosides, tetracyclines, fosfomycin and tigecycline. A major dissimilarity was polymyxin susceptibility in 6_GR_12 (sensitive in BMD, resistant in VITEK2) and resistance in 23_GR_13 (resistant in BMD, sensitive in VITEK2). Polymyxin resistance was identified in 19 of the isolates and minimum inhibitory concentrations (MICs) ranged from 8 to >64 mg l^−1^. An abundance of acquired resistance genes (Table 2) was detected and this presence corresponded to the antimicrobial testing phenotype. This analysis did not identify *mcr* genes (*mcr*-*1*, -*2*, -*3*, -*4*, -*5*) in these strains. Only 18_GR_14 and 19_GR_14 were not identified as extended-spectrum beta-lactamase producers amongst the polymyxin-resistant strains. Consequently, all polymyxin-resistant strains that harboured non-susceptibility to at least one antibiotic in 15 or more of the 17 antimicrobial categories were defined as XDR.

**Table 1.** BMD and VITEK2 antimicrobial testing for the 24 clinical isolates

Strain*	**Source**†	**Resistance profile**‡																						
		1			2	3	4		5	6			7	8	9	10	11	12	13	14	15	16	17	
		AMK	GEN	TOB	CPT	TZP	IPM	MEM	CFZ	FEP	CTX	CAZ	FOX	CIP	SXT	TGC	ATM	AMP	SAM	CHL	FOF	CST	MIN	TET
1_GR_13	St	R	R	R	R^N^	R	R	R	R^N^	R	R	R	R	R	R	R	R	R	R	R	R	R	R	R
2_GR_12	U	R	R	R	R^N^	R	R	R	R^N^	R	R	R	R	R	R	I^R^	R	R	R	R	R^S^	R	R	R
3_GR_13	S	R	R	R	R^N^	R	R	R	R^N^	R^I^	R	R	R	R	R	I	R	R	R	R	R	R^I^	I^R^	R
4_GR_12	B	R	R	R	R^N^	R	R	R	R^N^	R	R	R	R	R	R	I^R^	R	R	R	R	R	R	I^R^	R
5_GR_13	St	S	S	I	R^N^	R	R	R	R^N^	R	R	R	R	R	R	I^R^	R	R	R	R	R	R	R	R
6_GR_12	St	S	S	I^R^	R^N^	R	R	R	R^N^	R	R	R	R	R	R	R	R	R	R	R	R	S^R^	I^R^	R
7_GR_13	St	R	S	R	R^N^	R	R	R	R^N^	R	R	R	R	R	R	R	R	R	R	R	R	R	S^R^	S^I^
8_GR_13	St	R	R	R	R^N^	R	R	R	R^N^	R	R	R	R	R	R	R	R	R	R	R	R	R	R	R
9_GR_12	Br	I^R^	S	R	R^N^	R	R	R	R^N^	R	R	R	R	R	R	I^R^	R	R	R	R	R	R	I^R^	S^I^
10_GR_13	B	S^R^	S	R	R^N^	R	R	R	R^N^	R	R	R	R	R	R	I	R	R	R	R	R	R	S^R^	S^I^
11_BR_13	U	S	S	R^N^	R^N^	R	R	R	R^N^	R	R	R	R	R	R^N^	I	R	R	R	S^N^	R^N^	R	S^N^	S^N^
12_BR_13	Br	S	R^S^	I^N^	R^N^	R	R	R	R^N^	R^I^	R	R	R	R	R^N^	I^R^	R	R	R	R^N^	R^N^	R	I^N^	S^N^
13_GR_14	Br	I^R^	S	R	R^N^	R	R	R	R^N^	R	R^N^	R	R	R	R	S^R^	R	R	R	R	R	R	S^I^	S^I^
14_GR_14	U	I^R^	S^R^	R	R^N^	R	R	R	R^N^	R	R^N^	R	R	R	R	S^R^	R	R	R	R	R	R	S^R^	S^R^
15_GR_13	St	I^R^	S	R	R^N^	R	R	R	R^N^	R^I^	R^N^	R	R	R	R	I^R^	R	R	R	R	R	R	S^I^	S
16_GR_13	St	R	R	R	R^N^	R	R	R	R^N^	R	R^N^	R	R	R	R	R^I^	R	R	R	R	R	R	I^R^	R
17_GR_14	St	R	R	R	R^N^	R	R	R	R^N^	R	R^N^	R	R	R	R	I	R	R	R	R	R	R	S^R^	R
18_GR_14	St	I^R^	S	R	R^N^	R	R	R	R^N^	R	R	R	R	R	R	I	R	R	R	R	R	R	S^I^	S
19_GR_14	St	I^R^	S	R	R^N^	R	R	R	R^N^	R	R	R	R	R	R	R	R	R	R	R	R	R	I^R^	I^R^
20_GR_12	St	R	S	R	R^N^	R	R	R	R^N^	R^I^	R	R	R	R	R	R	R	R	R	R	R	S	R	R
21_GR_13	U	S	S^R^	I	R^N^	R	R	I^R^	R^N^	R^I^	R	R	R	S	R	S	S	R	R	S	S	S	S	S
22_GR_12	S	I^R^	S	R	R^N^	R	R	R	R^N^	R^I^	R	R	R	R	R	I	R	R	R	R	R^S^	S	S^I^	S
23_GR_12	St	R	R	R	R^N^	R	R	R	R^N^	R	R	R	R	R	R	R	R	R	R	R	R^S^	R^S^	R	R
24_GR_13	St	I^R^	S	R	R^N^	R	R	R	R^N^	R^I^	R	R	R	R	R	I^R^	R	R	R	R	R	S	I^R^	S

*Strain identification: numerical order catalogued at IMB_Country (GR, Greece; BR, Brazil)_last two digits of isolation year.

†Source represented as B, blood; Br; bronchial secretion; U, urine; S, sputum; St, stool.

‡Antibiotic resistance as determined by BMD according to CLSI guidelines [EUCAST for fosfomycin (disc diffusion) and tigecycline] and in superscript, any discrepancies identified in VITEK2 results. Antibiotic classes tested include: 1, aminoglycosides (amikacin, AMK; gentamicin, GEN; tobramycin, TOB); 2, anti-methicillin-resistant *Staphylococcus aureus* (MRSA) cephalosporins (ceftaroline, CPT); 3, anti-pseudomonal penicillins + β-lactamase inhibitors (piperacillin-tazobactam, TZP); 4, carbapenems (imipenem, IPM; meropenem, MEM); 5, non-extended spectrum cephalosporins (1st and 2nd generation) (cefazolin, CFZ); 6, extended-spectrum cephalosporins (3rd and 4th generation) (cefepime, FEP; cefotaxime, CTX, ceftazidime, CAZ); 7, cephamycins (cefoxitin, FOX); 8, fluoroquinolones (ciprofloxacin, CIP); 9, folate pathway inhibitors (trimethoprim-sulfamethoxazole, SXT); 10, glycylcyclines (tigecycline, TGC); 11, monobactams (aztreonam, ATM); 12, penicillins (ampicillin, AMP); 13, penicillins + β-lactamase inhibitors (amipicillin-sulbactam, SAM); 14, phenicols (chloramphenicol, CHL); 15, phosphonic acids (fosfomycin, FOF); 16, polymyxins (colistin, CST); 17, tetracyclines (minocycline, MIN; tetracycline, TET). R, resistant; I, intermediate; S, susceptible; N, not tested.

**Table 2.** Potential mutations contributing to polymyxin resistance and acquired resistance genes

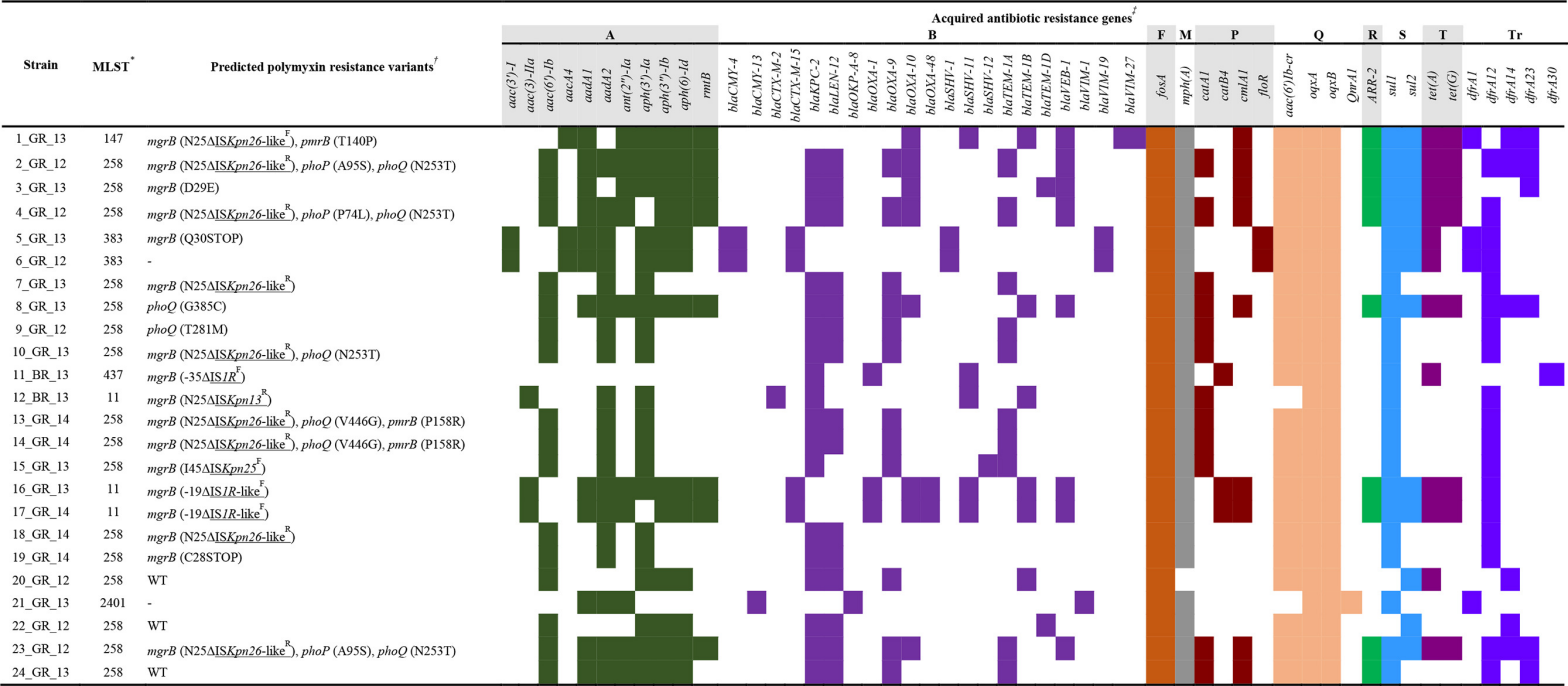

*Multilocus sequence type as identified through MultiLocus Sequence Typing Server 1.8.

†Variations detected in *mgrB*, *phoPQ* and *pmrAB* potentially causing polymyxin resistance. Significant non-synonymous changes determined by PROVEAN analysis. WT (wild-type) alleles in comparison to 20_GR_12. Displayed as gene impacted, initial amino acid, position and new amino acid. If a dash (–) is shown in front of the position, variant is encoded upstream and if a dash (–) is only displayed, no significant non-synonymous changes were detected in these loci. Insertion sequences (underlined) classified as Δ, identity as per ISFinder and orientation in superscript. Orientation determined as forward, ^F^, if transposase is in the same direction as *mgrB* and conversely, reverse, ^R^, if in the opposite direction to *mgrB*.

‡Acquired antibiotic resistance genes detected via ResFinder 3.0. Classes of antibiotics impacted are displayed as: A, aminoglycoside; B, beta-lactam; F; fosfomycin; M, macrolide; P, phenicol; Q, quinolone; R, rifampicin; S, sulphonamide; T, tetracycline; Tr, trimethoprim. Shading indicates detection of a gene (≥90 % homology, ≥60 % sequence length).

### Sequence type determination

**Fig. 1.**
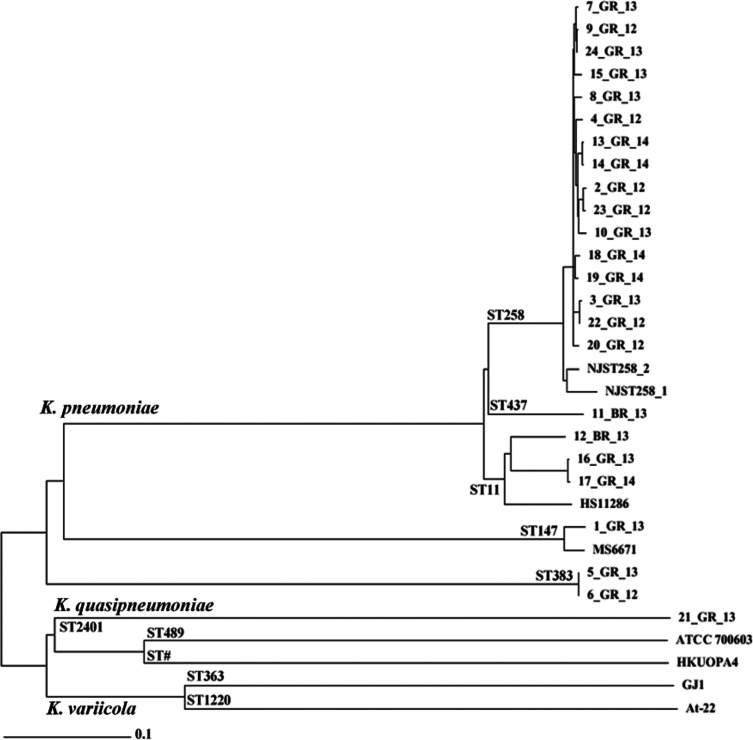
Neighbour-joining tree of core genome MLST of 24 *Klebsiella* clinical isolates. Clustering of STs is indicated at the base of diverging branches. ST# indicates an uncharacterized MLST according to MLST server 1.8. cgMLST was used to compare completed assemblies including HS11286 (ST11), MS6671 (ST147), and NJST258_1 and NJST258_2 (ST258). Assemblies were also compared against *K. quasipneumoniae* (ATCC 700603, HKUOPA4) and *K. variicola* (At-22, GJ1) genomes.

Sixteen of the 22 Greek clinical strains were found to belong to ST258 and the remaining were ST11, ST147 or ST383 (Table 1). While 5_GR_13 and 6_GR_12 were both ST383, only 5_GR_13 was resistant to polymyxin. Among the two strains from Brazil, 11_BR_13 was ST437 and 12_BR_13 was ST11. Strain 21_GR_13 had a profile previously undefined and has been newly designated ST2401. Further cgMLST studies were conducted on the isolates using complete assemblies of reference genomes for ST11 (HS11286), ST147 (MS6671), ST258 (NJST258_1, NJST258_2), *K. quasipneumoniae* (KQ) (ATCC 700603, HKUOPA4) and *K. variicola* (KV) (At-22, GJ1) (Fig. 1). For the ST258 isolates, these were more similar to NJST258_2 than to NJST258_1. Within this cluster, 7_GR_13, 9_GR_12 and 24_GR_13 were closely related (≤15 allelic changes). Similarly grouped together were 2_GR_12 and 23_GR_12; 3_GR_13 and 22_GR_12; 13_GR_14 and 14_GR_14; and 18_GR_14 and 19_GR_14. In ST11, 16_GR_13 and 17_GR_14 harboured only three allele differences and the Brazilian isolate, 12_BR_13, had 206 variants apparent. ST383 isolates 5_GR_13 and 6_GR_12 only exhibited one allele change. ST147 1_GR_13 was not clonal to the previous pandrug-resistant KP, MS6671. Clustering analysis revealed 21_GR_13 as KQ rather than KV.

### MgrB disruption

Seventeen of the 19 polymyxin-resistant strains exhibited either missense mutations, nonsense mutations or IS elements in *mgrB* (Table 2). Both 5_GR_13 and 19_GR_14 harboured a truncation while an amino acid change, D29E, was apparent in 3_GR_13. IS element disruptions in *mgrB* were present in 14 strains and in nine isolates, and an IS*5*-like element was integrated at nucleotide position 75 (Fig. S1). Sanger sequencing revealed this element was closely related to IS*Kpn26*, herein known as IS*Kpn26*-like, except for 12_BR_13 which matched IS*Kpn13.* IS*1R* was detected upstream of *mgrB* in 11_BR_13 and an IS*1R*-like (A>C, 393 bp; C>T, 396 bp) element in 16_GR_13 and 17_GR_14. Strain 15_GR_13 had a deletion of the *mgrB* locus from nucleotide position 133 onwards. The 127 bp flanking region mapped to IS*Kpn25* with the transposase in the same orientation as *mgrB*. All three of the IS*1* element insertions, but only one of the eight IS*Kpn26*-like element insertions had the transposase in the same orientation as *mgrB*.

### Single, multiple and heterogeneous mutations

Mutations in genes commonly identified to confer polymyxin resistance in KP include *mgrB*, *phoPQ* and *pmrAB* (Table 2). Several non-synonymous mutations were identified across the isolates (Table S3). These mutations were analysed through the prediction tool, PROVEAN, and not all were identified to be deleterious, although this does not negate a functional purpose for these variants. ST383 contained several mutations in *pmrAB* although only Q30STOP in polymyxin-resistant 5_GR_13 was predicted to have an impact. Similarly, neutral changes in all four of these genes were detected in polymyxin-susceptible KQ strains ATCC 700603 and 21_GR_13. Strains 8_GR_13 and 9_GR_12 harboured a single detrimental missense mutation in *phoQ*. Seven of the 17 isolates containing an *mgrB* variant were accompanied by one or more missense mutations in *phoPQ* and/or *pmrB*. Predicted deleterious variants segregating with disrupted *mgrB* included *pmrB* (T140P, P158R), *phoP* (P74L, A95S) and *phoQ* (N253T, V446G), which were commonly found in the ST258 lineage. V446G (*phoQ*) and P158R (*pmrB*) were heterogeneous in 13_GR_14 [mutation allele frequency of 65 % (V446G) and 66 % (P158R)] and 14_GR_14 [mutation allele frequency of 52 % (V446G) and 57 % (P158R)]. Assembly revealed 23_GR_12 harboured an IS*Kpn26-*like disrupted *mgrB* alongside the intact version with mutations in *phoP* and *phoQ* in 57 % of the samples. Furthermore, assemblies for *mgrB*, *pmrAB* and *phoPQ* were aligned to ATCC 700603 (Table S4). Several non-synonymous mutations were detected, but the majority were not predicted to be deleterious. Various mutations were unique to KP compared to KQ. ST11, 147, 258 and 437 remained conserved across these genes with the exception of mutations predicted to be deleterious. ST383 harboured several dissimilarities including the lack of *pmrA* (D131N) and *pmrB* (S105N) and gain of *pmrA* (G144D, D149E) and *pmrB* (A5V, M175V). Only subtle differences were observed in KQ isolate 21_GR_ 13, which included *pmrA* (I220N, D221E) and *pmrB* (G358A). Predicted deleterious mutations detected both in polymyxin-susceptible and in polymyxin-resistant isolates included *pmrA* (Q140L) and *pmrB* (R256G).

### Role of *mgrB* disruptions and presence of heterogeneity via complementation assays

**Fig. 2.**
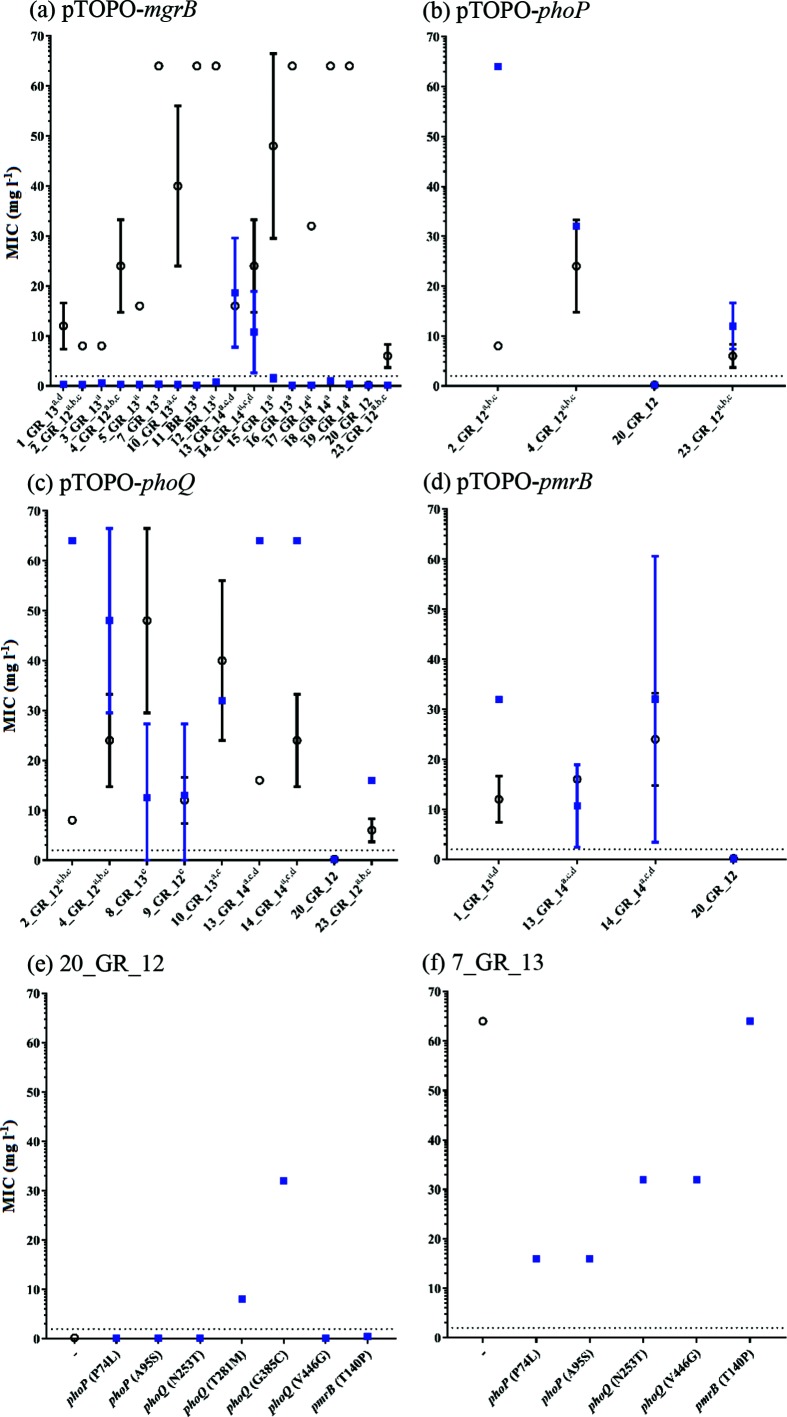
Complementation assays and influence of gene on polymyxin resistance. Polymyxin B MICs measured before (**○**) and after (■) complementation of the wild-type gene (a) pTOPO-*mgrB*, (b) pTOPO-*phoP*, (c) pTOPO-*phoQ* or (d) pTOPO-*pmrB* in the indicated resistant isolates. (e) Mutated genes complemented into 20_GR_12 (polymyxin-susceptible isolate) to determine if the variant induces polymyxin resistance. (f) Complementation of 7_GR_13 (IS element disrupted *mgrB* control) to detect potential suppressor mutations. Strains shown on the *x-*axis for (a)–(d) and superscript indicates variants in genes including *mgrB* (a), *phoP* (b), *phoQ* (c) and *pmrB* (d) that differ from 20_GR_12. For (e) and (f), the *x*-axis shows the gene complemented with amino acid variation in parentheses. The dotted line at 2 mg l^−1^ represents the breakpoint for polymyxin B. Values are mean±sd, where no error bar indicates no fluctuation in MIC (*n*≥2 colonies).

Complementation of the WT gene elucidated the role of these mutations in resistance (Fig. 2). MICs were determined against polymyxin B and colistin, but no difference was observed. Introduction of pTOPO-*mgrB* restored susceptibility in all resistant isolates with *mgrB* coding mutations or upstream disruptions, with the exception of two strains heterogeneous for the *mgrB* disruption and a *pmrB* coding mutation (13_GR_14 and 14_GR_14) (Fig. 2a). For these two strains, pTOPO-*mgrB* restored susceptibility in none of three 13_GR_14 colonies and one of three 14_GR_14 colonies. Transformation of one out of three colonies for both 13_GR_14 and 14_GR_14 strains with pTOPO-*pmrB* restored susceptibility (Fig. 2d) and *mgrB* amplification of these colonies revealed an intact *mgrB* locus (Fig. S2). Colonies which were reverted on complementation were further passaged three times with no antibiotic pressure in order to remove the plasmid and discern if these mutations were contributing to resistance. After passaging, pTOPO-*mgrB* isolates harboured an MIC of ≥64 mg l^−1^ whilst pTOPO-*pmrB* colonies had an MIC of 16 mg l^−1^, confirming two resistant populations in these samples. 23_GR_12 was also observed to have a heterogeneous *mgrB* disruption but did not carry a corresponding *pmrB* mutation, although it harboured similar mutations to 2_GR_12 in *phoPQ*. Amplification of *mgrB* identified two of three 23_GR_12 transformed colonies contained the IS element disruption and reverted to being susceptible upon complementation with pTOPO-*mgrB*.

### Validation of resistance-conferring mutations in *phoQ*

Strains 8_GR_13 and 9_GR_12 harboured a single mutation in *phoQ* potentially conferring resistance (Table 2). When these isolates were transformed with pTOPO-*phoQ*, results remained variable where a lack of growth was present in a susceptible range (MIC ≤2 mg l^−1^), although several wells containing high polymyxin B concentrations exhibited growth (Fig. 2c). This result was reproducible (*n*≥4) and therefore the mutated gene was introduced into a polymyxin-susceptible isolate, 20_GR_12 (Fig. 2e). This complementation resulted in a consistent polymyxin-resistant phenotype.

### Potential suppressor mutations in *phoPQ*

Several mutations co-segregating with the IS element-disrupted *mgrB* were detected, including *phoP* (P74L, A95S), *phoQ* (N253T, V446G) and *pmrB* (T140P). Complementation of WT genes in these isolates facilitated a ≥2-fold increase in MIC with the exception of 10_GR_13, which had an additional predicted neutral mutation in *phoQ* (A225T) (Table S3, Fig. 2b–d). To evaluate the potential influence of these mutations on polymyxin resistance, mutated genes were introduced into a strain only containing the *mgrB* IS element disruption, 7_GR_13 (Fig. 2f). Complementation of mutant *pmrB* (T140P) into 7_GR_13 did not lead to an observable corresponding reduction in MIC, but once transformed into 20_GR_12, a twofold increase in MIC was apparent (Fig. 2e). Variants in *phoQ* (N253T and V446G) exhibited a twofold reduction in MIC (Fig. 2e). Initially, the *phoQ* (V446G) mutation was anticipated to segregate with the *mgrB-*disrupted population in 13_GR_14 and 14_GR_14, but when *phoQ* was amplified from a colony reverted to susceptible via pTOPO-*mgrB* complementation, the WT *phoQ* was observed (Fig. S3). The *phoQ* (V446G) mutation was successfully amplified from a 14_GR_14 colony containing the *pmrB* (T158R) mutation. Although this mutation did not segregate with disrupted *mgrB*, it may act as a partial suppressor mutation when a resistance-conferring mutation is present in *pmrB*. Interestingly, a ≥4-fold reduction in MIC was witnessed for *phoP* mutations P47L and A95S, indicating partial suppressor mutations (Fig. 2e).

## Discussion

Polymyxin resistance in XDR-KP is of grave concern given that this is a last-line antibiotic, and resistance is increasingly prevalent in countries such as Greece and Brazil [10, 12–14, 49]. We evaluated the genetic basis of polymyxin resistance in a series of Greek and Brazilian clinical isolates from patients in 2012–2014 and found variants in genes *mgrB*, *phoPQ* and *pmrAB*. Causative mutations attributed to polymyxin resistance were identified in these loci, but the contribution of other genes warrants further investigation.

Inactivation of *mgrB* was highly prevalent in these strains with an IS*Kpn26*-like element being the predominant cause of resistance, as confirmed by complementation restoring susceptibility in all isolates. Several other studies have observed an IS*5*-like element integration in the same position, including reports from Greece, Italy, France, Turkey and Colombia [8, 9, 50, 51]. The IS*Kpn26*-like element resembled the same sequence from Greek isolates previously described [51]. We identified that this mutation still persisted in 2014, after being first detected in 2012 [9]. Disruptions in *mgrB* including the IS*Kpn26*-like forward insertion at nucleotide 75 in ST147, IS*Kpn13* integration at nucleotide 75 in ST11 and IS*Kpn25* in the ST258 lineage have yet to be reported. We identified IS*1R* or IS*1R*-like elements positioned upstream of *mgrB* in three isolates (11_BR_13, 16_GR_13, 17_GR_14) which were reverted upon complementation indicating an impact on the promoter region.

Truncations identified at positions 28 and 30 of *mgrB* have been previously detected, although these were identified in differing STs, indicating mutations potentially have arisen independently in Greece [24, 52]. Complementation restored susceptibility to polymyxins for these mutations and this study further revealed the amino acid change D29E in 3_GR_13 to be a resistance-conferring mutation. These findings support the notion that intact MgrB is required to confer negative feedback on PhoPQ [8]. The inactivation of *mgrB* is prevalent in polymyxin-resistant KP and may arise owing to its capacity to promote virulence and further attenuate the early host defence response, with little or no fitness cost [53].

Single predicted detrimental mutations were observed in the *phoQ* histidine kinase region, critical for phosphorylation and interaction with *phoP*, in 8_GR_13 (G385C) and 9_GR_12 (T281M). The G385C mutation had previously been reported, [24], but in a different ST. Complementation revealed an inconsistent MIC for these strains, although when a polymyxin-susceptible isolate was transformed with the mutated gene, full resistance was restored. Dominance of mutated *phoQ* has recently been highlighted and these results may imply the inability of pTOPO-*phoQ* to override the resistance caused by these mutations [54]. Furthermore, the inconsistencies in MIC may be attributed to the heightened expression of WT *phoQ* in the pCR-Blunt II-TOPO vector and warrants further investigation.

Several non-synonymous changes were identified to be not deleterious according to PROVEAN analysis. Notably, these were abundant in KQ strains, including 21_GR_13 and KP ST383 isolates. When these clinical isolates were aligned to ATCC 700603, multiple coding changes were identified, with the majority detected as neural changes with the exception of *pmrA* (Q140L) and *pmrB* (R256G). These mutations represent lineage-specific mutations, but this does not negate the possibility of previously resistance-conferring variants being acquired in these loci with subsequent reversion mutations to give rise to a susceptible phenotype.

Heterogeneity was apparent in several isolates. In near equal ratios, 13_GR_14 and 14_GR_14 possessed the IS*Kpn26*-like *mgrB* disruption and a new mutation conferring resistance in *pmrB*, P158R, as determined by complementation. 23_GR_12 contained approximately half the reads mapping to the undisrupted genes and the other to the IS*Kpn26*-like strain, with several additional predicted deleterious mutations. This heterogeneity may explain the initial clinical detection for this isolate to be polymyxin-susceptible.

Several isolates harbouring IS*Kpn26*-like element-disrupted *mgrB* were accompanied by mutations in *phoPQ* and/or *pmrB*. These changes were present in ≥98 % of reads, making the involvement of heterogeneity unlikely. Once complemented, an increase in resistance was commonly recorded. This potentially reflects partial suppressor mutations as strains which solely possessed this IS element disruption commonly exhibited a heightened MIC of ≥64 mg l^−1^. One variant segregating with this disruption included *pmrB* T140P. This had formerly been identified in an ST258 lineage but even when the resistant gene was complemented, the MIC increased by twofold but was not defined as clinically resistant [24, 55].

When mutated *phoP* or *phoQ* were introduced into the *mgrB-*disrupted isolate, a reduction in MIC was apparent. The involvement of additional mutations in PhoPQ causing a suppressing effect on the level of resistance in a background where the disrupted *mgrB* is lacking has yet to be reported in KP. Previous research by Miller *et al.* [56] determined additional mutations in PhoPQ altered polymyxin resistance in *Pseudomonas aeruginosa*. Their study describes *phoP* mutations with the capacity to partially or fully suppress resistance-causing mutations in *phoQ*. These mutations in *phoP* were near or within the DNA binding site, which differs from our results, where the mutations are impacting the response regulatory region that interacts with PhoQ. Conversely, all mutations partially suppressing the MIC were identified to be targeting the HAMP (present in Histidine kinases, Adenylate cyclases, Methyl-accepting proteins and Phosphatases) domain and histidine kinase component of PhoQ. These were in regions similar to revertant *P. aeruginosa* strains identified by Lee and Ko [57]. We postulate these mutations are perturbing the critical transfer of phosphoryl groups from the histadine kinase of PhoQ to PhoP and subsequent *pmrD* expression. Whether these mutations constitute a fitness advantage due to the reduction of metabolism required for the production of lipopolysaccharide modifications is yet to be discerned. Furthermore, due to variability in some of the complementation data, a knockout *phoPQ* background and introduction of genes that are potential suppressor mutations is required.

Rapid and accurate detection of mutations attributed to polymyxin resistance remains a long-standing problem. Our research has contributed to the current understanding of the dissemination and evolution of this resistance in KP. Although our sample size is limited, this study highlights several issues arising from solely interrogating genomes for resistance detection, including ST-specific non-synonymous changes, and heterogeneity. Our study reveals several mutations causing polymyxin resistance across various STs in comparison with prior literature. These include the *mgrB* IS*Kpn26*-like disruption (nucleotide 75), truncations in *mgrB* (nucleotides 28 and 30) and a missense mutation in *phoQ* (G385C). The study provides the first potential report of suppressor mutations for polymyxin resistance. Through complementation assays, we have discerned the role of these modifications and have identified resistance-causing variants that can be monitored in future genome-based diagnostics.

## Data bibliography

1. NCBI Bioproject PRJNA307517 (2016).
2. **Liu P, Li P, Jiang X, Bi D, Xie Y, Tai C, *et al.*** Complete genome sequence of *Klebsiella pneumoniae* subsp*. pneumoniae* HS11286, a multidrug-resistant strain isolated from human sputum. *J Bacteriol* 2012;194:1841–1842. NCBI BioProject PRJNA78789.
3. **Zowawi HM, Forde BM, Alfaresi M, Alzarouni A, Farahat Y, *et al.*** Stepwise evolution of pandrug-resistance in *Klebsiella pneumoniae*. *Sci Rep* 2015;5:15082. European Nucleotide PRJEB7538.
4. **Deleo FR, Chen L, Porcella SF, Martens CA, Kobayashi SD, Porter AR, *et al.*** Molecular dissection of the evolution of carbapenemresistant multilocus sequence type 258 *Klebsiella pneumoniae. Proc Natl Acad Sci USA* 2014;111:4988–4993. European Nucleotide PRJNA237670.
5. **Elliott AG, Ganesamoorthy D, Coin L, Cooper MA, Cao MD**. Complete Genome Sequence of *Klebsiella quasipneumoniae* subsp. *similipneumoniae* Strain ATCC 700603. *Genome Announc* 2016;4:e00438-16.
6. **Liu L, Ahmad AH, Leung FC**. *Klebsiella quasipneumoniae* strain HKUOPA4, complete genome. NCBI Bioproject PRJNA224116 (2017).
7. **Pinto-Tomás AA, Anderson MA, Suen G, Stevenson DM, Chu FS, *et al***. Symbiotic nitrogen fixation in the fungus gardens of leaf-cutter ants. *Science* 2009;326:1120–1123.
8. **Di DY, Jang J, Unno T, Hur HG**. Emergence of *Klebsiella variicola* positive for NDM-9, a variant of New Delhi metallo-β-lactamase, in an urban river in South Korea. *J Antimicrob Chemother* 2017;72:1063–1067.

## Supplementary Data

1269 Supplementary File 1
