# Supplementary material for: Multifactorial chromosomal variants regulate polymyxin resistance in extensively drug-resistant Klebsiella pneumoniae
Source: Microb Genom. 2018 Feb 12;4(3):e000158. doi: 10.1099/mgen.0.000158 (PMC5885010; doi:10.1099/mgen.0.000158)
Supplement: Supplementary File 1 [file mgen-4-158-s001.pdf]

Multifactorial Chromosomal Variants Regulate Polymyxin Resistance in Extensively Drug-Resistant *Klebsiella pneumoniae*

SUPPLEMENTARY FIGURES AND TABLES

Table S1. Oligonucleotides used in this study

Assay	Gene	Forward sequence (5’ to 3’)	Reverse sequence (5’ to 3’)	Reference
PCR				
	mgrB	ATT CTG CCG CTT TTG CTG	CGT TTT GAA ACA AGT CGA TGA	24
Complementation				
	mgrB	TTA AGA AGG CCG TGC TAT CC	AAG GCG TTC ATT CTA CCA CC	48
	phoP	CAC CAG GGG CCC TTT TTA T	GCT AAC GCT ATA GCC CAC CA	This study
	phoQ	ATA CCC ACA GGA CGT CAT CA	CAG GTG TCT GAC AGG GAT TA	48
	pmrB	ACC TAC GCG AAA AGA TTG GC	GAT GAG GAT AGC GCC CAT GC	48

Table S2. Broth microdilution values acquired for the 24 clinical isolates

Strain*	Resistance Profile†																						
	1			2	3	4		5	6			7	8	9	10	11	12	13	14	15	16		17
	AMK	GEN	TOB	CPT	TZP	IPM	MEM	CFZ	FEP	CTX	CAZ	FOX	CIP	SXT	TGC	ATM	AMP	SAM	CHL	FOF	CST	PMB	MIN	TET
ATCC 25922	≤2	≤0.5	≤0.5	≤0.25	≤1/4	≤0.5	≤0.25	≤2	≤0.5	≤0.25	≤0.5	≤2	≤0.125	≤0.5/ ≤16	≤0.25	≤0.5	8	8/4	≤8	≥27	0.25	0.25	≤0.5	≤0.5
ATCC 700603	≤1	≤8	≤8	≤4	128/> 64	≤0.5	≤0.25	≥64	≤0.5	2	64	≤16	≤0.125	≤2/> 128	≥4	≥32	>64	>64/ ≥32	≥32	≤12	0.5	0.5	≥16	8
ATCC 13883	≤0.5	≤0.5	≤0.5	≤0.25	16/4	≤0.5	≤0.25	≤2	≤0.5	≤0.25	≤0.5	≤2	≤0.125	≤0.5/ ≤32	≤1	≤0.5	>64	>64/ ≥8	≤4	≤18	≤4	≤4	≤1	≤0.5
ATCC BAA-2146	>64	>64	>64	>32	>128/ >64	>32	>32	>64	>64	>32	>64	>64	>16	>64/ >128	≥4	>64	>64	>64/ ≥32	>64	≤20	≤0.25	≤0.25	≥64	>64
1_GR_13	>64	>64	>64	>32	>128/ >64	≥64	>32	>64	>64	>32	>64	>64	>16	>64/ 128	≥2	>64	>64	>64/ >64	64	≤19	16	≥8	≥16	>64
2_GR_12	>64	>64	>64	>32	>128/ >64	≥64	>32	>64	>64	>32	>64	>64	>16	>64/ 128	2	>64	>64	>64/ >64	>64	≤20	16	8	16	>64
3_GR_13	>64	>64	>64	>32	>128/ >64	≥16	≥8	>64	≥16	>32	>64	64	>16	>64/ 128	2	>64	>64	>64/ >64	≥32	≤22	16	8	≥8	>64
4_GR_12	>64	>64	>64	>32	>128/ >64	>64	>32	>64	>64	>32	>64	>64	>16	>64/ 128	2	>64	>64	>64/ >64	>64	20	32	≥16	≥8	>64
5_GR_13	≤16	≤4	≥8	>32	>128/ >64	≥32	≥4	>64	>64	>32	>64	>64	>16	>64/ 128	2	>64	>64	>64/ >64	>64	≤21	≥16	16	16	>64
6_GR_12	≤8	2	8	>32	>128/ >64	32	≥8	>64	≥64	>32	>64	>64	>16	>64/ 128	≥2	>64	>64	>64/ >64	>64	20	≤0.5	≤1	≤8	>64
7_GR_13	64	≤2	64	>32	>128/ >64	≥64	>32	>64	>64	>32	>64	>64	>16	>64/ 128	≥2	>64	>64	>64/ >64	>64	19	>64	≥64	4	≤4
8_GR_13	>64	>64	>64	>32	>128/ >64	>64	>32	>64	>64	>32	>64	>64	>16	>64/ 128	≥2	>64	>64	>64/ >64	>64	≤19	64	≥32	≥16	>64
9_GR_12	≥32	≤2	≥32	>32	>128/ >64	16	≥4	>64	>64	>32	>64	64	>16	>64/ 128	2	>64	>64	>64/ >64	>64	≤8	≥16	≥8	≤8	≤4
10_GR_13	16	≤1	≥16	>32	>128/ >64	>64	>32	>64	>64	>32	>64	>64	>16	>64/ 128	2	>64	>64	>64/ >64	>64	≤20	64	≥32	≤4	≤4
11_BR_13	≤16	≤0.5	≥16	>32	>128/ >64	>64	>32	>64	≥64	>32	≥64	>64	>16	>64/ 128	≤2	>64	>64	>64/ >64	8	≤15	≥64	64	≤4	≤2
12_BR_13	≤1	>64	≥8	>32	>128/ >64	≥64	>32	>64	>64	>32	≥64	>64	>16	>64/ 128	≤2	>64	>64	>64/ >64	>64	23	>64	>64	≤8	≤2
13_GR_14	≤32	≤0.5	≥16	>32	>128/ >64	>64	>32	>64	>64	>32	>64	>64	>16	>64/ 128	≤1	>64	>64	>64/ >64	>64	20	16	16	≤1	≤0.5
14_GR_14	≤32	≤0.5	≥16	>32	>128/ >64	>64	>32	>64	>64	>32	>64	>64	>16	>64/ 128	≤1	>64	>64	>64/ >64	>64	20	≥32	≥16	≤2	≤1
15_GR_13	≤32	≤1	≥16	>32	>128/ >64	32	≥8	>64	≥64	>32	>64	64	>16	>64/ 128	2	>64	>64	>64/ >64	>64	≤22	64	≥32	≤4	1

16_GR_13	>64	>64	>64	>32	>128/ >64	≥8	≥16	>64	>64	>32	>64	64	>16	>64/ 128	≥2	>64	>64	>64/ >64	32	≤19	64	64	≤8	>64
17_GR_14	>64	>64	>64	>32	>128/ >64	≥4	≥4	>64	>64	>32	>64	32	>16	>64/ 128	2	>64	>64	>64/ >64	≥16	≤20	64	32	4	>64
18_GR_14	≤32	≤0.5	≥16	>32	>128/ >64	>64	>32	>64	>64	>32	>64	>64	>16	>64/ 128	2	>64	>64	>64/ >64	≥32	≤20	64	64	2	≤2
19_GR_14	≤32	≤0.5	≥16	>32	>128/ >64	≥16	≥8	>64	>64	>32	>64	>64	>16	>64/ 128	≥4	>64	>64	>64/ >64	64	21	64	64	≥8	8
20_GR_12	64	1	64	>32	>128/ >64	≥8	≥8	>64	≥16	>32	>64	64	>16	>64/ 128	≥2	>64	>64	>64/ ≥64	≥32	≤22	0.125	≤0.25	16	>64
21_GR_13	≤4	≤4	≥8	>32	>128/ >64	8	≤2	>64	≥16	>32	>64	>64	0.25	>64/ 128	1	≤4	>64	>64/ ≥64	≤4	≥24	≤0.25	0.125	≤1	≤0.5
22_GR_12	≤32	≤1	≥16	>32	>128/ >64	≥16	≥8	>64	≥16	>32	>64	64	>16	>64/ 128	≥1	>64	>64	>64/ ≥64	32	20	≤0.25	≤0.25	≤4	1
23_GR_12	>64	>64	>64	>32	>128/ >64	≥64	>32	>64	>64	>32	>64	>64	>16	>64/ 128	8	>64	>64	>64/ >64	>64	≤20	≥8	≥4	>64	>64
24_GR_13	≤32	≤1	≥16	>32	>128/ >64	≥8	≥4	>64	≥16	>32	>64	≥32	>16	>64/ 128	2	>64	>64	>64/ ≥64	>64	≤23	≤0.25	≤0.25	≤8	2

*Strain identification, numerical order catalogued at IMB_Country (GR:Greece, BR:Brazil)_last two digits of isolation year.

†Antibiotic resistance as determined by broth microdilution according to CLSI guidelines except fosfomycin (disk diffusion) and tigecycline which followed EUCAST breakpoints. Antibiotic classes tested include **1**, Aminoglycosides (Amikacin, AMK; Gentamicin, GEN; Tobramycin, TOB); **2**, Anti-MRSA cephalosporins (Ceftaroline, CPT); **3**, Antipseudomonal penicillins + β-lactamase inhibitors (Piperacillin-tazobactam, TZP); **4**, Carbapenems (Imipenem, IPM; Meropenem, MEM); **5**, Non-extended spectrum cephalosporins (1st and 2nd generation) (Cefazolin, CFZ); **6**, Extended-spectrum cephalosporins (3rd and 4th generation) (Cefepime, FEP; Cefotaxime, CTX, Ceftazidime, CAZ); **7**, Cephamycins (Cefoxitin, FOX); **8**, Fluoroquinolones (Ciprofloxacin, CIP); **9**, Folate pathway inhibitors (Trimethoprim-sulfamethoxazole, SXT); **10**, Glycylcyclines (Tigecycline, TGC); **11**, Monobactams (Aztreonam, ATM); **12**, Penicillins (Ampicillin, AMP); **13**, Penicillins + β-lactamase inhibitors (Amipicillin-sulbactam, SAM); **14**, Phenicols (Chloramphenicol, CHL); **15**, Phosphonic acids (Fosfomycin, FOF); **16**, Polymyxins (Colistin, CST;

Polymyxin B, PMB); **17**, Tetracyclines (Minocycline, MIN; Tetracycline, TET). The two antibiotics for TZP, SXT and SAM were assayed separately and both MICs separated with a /. Shading indicates R, Resistant; I, Intermediate; S, Susceptible.

Table S3. Non-synonomous changes relative to 20_GR_12 and PROVEAN prediction

Strain*	Polymyxin Profile†	Gene	Variant‡	PROVEAN score§
ATCC 700603	S	*pmrA*	S64A, N131D, L140Q, E199D, N219H	-
		pmrB	T8N, N105S, A228T, Q232E, I242V, N244S, G256R, E272Q, Q356R	-
		phoP	R34K	-
		phoQ	Q92K, A106T, E112D, I139V, L163F, V196I, T372S, Q424P, Q482L, Q487E	-
1_GR_13$^{\Delta}$	R	*pmrB*	T140P	-4.39
2_GR_12$^{\Delta}$	R	*phoP*	A95S	-2.97
		phoQ	N253T	-3.86
3_GR_13	R	*mgrB*	D29E	-3.97
4_GR_12$^{\Delta}$	R	*phoP*	P74L	-9.98
		phoQ	N253T	-3.86
5_GR_13	R	*mgrB*	Q30STOP	-14.86
		pmrA	N131D, G144D, D149E, N219H	-
		pmrB	A5V, N105S, M175V, A228T, G256R	-
6_GR_12	S	*pmrA*	N131D, G144D, D149E, N219H	-
		pmrB	A5V, N105S, M175V, A228T, G256R	-
8_GR_13	R	*phoQ*	G385C	-8.06
9_GR_12	R	*phoQ*	T281M	-5.36
10_GR_13$^{\Delta}$	R	*phoQ*	A225T, N253T	-, -3.86
13_GR_14$^{\Delta}$	R	*pmrB*	P158R (66)	-8.68
		phoQ	V446G (65)	-5.46
14_GR_14$^{\Delta}$	R	*pmrB*	P158R (57)	-8.68
		phoQ	V446G (52)	-5.46
19_GR_14	R	*mgrB*	C28STOP	-19.00
21_GR_13	S	*pmrA*	S64A, N131D, L140Q, E199D, N219H, I220N, D221E	-
		pmrB	T8N, N105S, A228T, Q232E, I242V, N244S, G256R, E272Q, Q356R, G358A	-
		phoP	R34K	-
		phoQ	Q92K, A106T, E112D, I139V, L163F, V196I, T372S, Q424P, Q482L, Q487E	-
23_GR_12$^{\Delta}$	R	*phoP*	A95S (57)	-2.97
		phoQ	N253T (57)	-3.86

*Strains also harboring an IS element disruption in *mgrB* shown as, $^{\Delta}$.

†S, Susceptible (MIC: ≤2 µg/ml); R, Resistant (MIC: >2 µg/ml).

‡Variant represented as initial amino acid, position and new amino acid; () corresponds to percentage of reads mapping to alteration if ≤98%.

§PROVEAN predicted deleterious score which is set to ≤-2.5; -, value not predicted deleterious.

Table S4. Non-synonymous mutations relative to ATCC 700603

MLST[*]	Gene[†]				
	mgrB	*phoP*	*phoQ*	*pmrA*	*pmrB*
11 (3)	-	K34R	K64R, K92R,T106A, D112E,V139E, F163L, I196V, S372T, P424Q, L482Q, E487Q	A64S, D131N, **Q140L**, D199E, H219N	N8T, S105N, T228A, E232Q, V242I, S244N,**R256G**, Q272E, R356Q,
147 (1)	-	K34R	K64R, K92R,T106A, D112E,V139E, F163L, I196V, S372T, P424Q, L482Q, E487Q	A64S, D131N, **Q140L**, D199E, H219N	N8T, S105N, **T140P**, T228A, E232Q, V242I, S244N,**R256G**, Q272E, R356Q,
258 (16)	**C28STOP**, **D29E**	K34R, **P74L**, **A95S**	K64R, K92R,T106A, D112E,V139E, F163L, I196V, **A225T**, **N253T**, **T281M**, **G385C** S372T, P424Q, **V446G**, L482Q, E487Q	A64S, D131N, **Q140L**, D199E, H219N	N8T, S105N, **P158R**,T228A, E232Q, V242I, S244N,**R256G**, Q272E, R356Q
383 (2)	**Q30STOP**	K34R	K64R, K92R,T106A, D112E,V139E, F163L, I196V, S372T, P424Q, L482Q, E487Q	A64S, **Q140L**, G144D,D149E, D199E	A5V, N8T,M175V, E232Q, V242I, S244N, Q272E, R356Q
437 (1)	-	K34R	K64R, K92R,T106A, D112E,V139E, F163L, I196V, S372T, P424Q, L482Q, E487Q	A64S, D131N, **Q140L**, D199E, H219N	N8T, S105N, T228A, E232Q, V242I, S244N,**R256G**, Q272E, R356Q
2401 (1)	-	-	-	I220N, D221E	G358A

[*]Multi-locus sequence type and in brackets, the number of isolates in this group.

[†]Genes *mgrB*, *pmrAB* and *phoPQ* were aligned to ATCC 700603 and represented as initial amino acid, position and new amino acid. Bold indicates a predicted deleterious variant in polymyxin-resistant strains according to PROVEAN analysis and an underline identifies this change also being present in polymyxin-susceptible isolates. Strains impacted by an IS element disruption in *mgrB* only had flanking regions interrogated to detect variants.

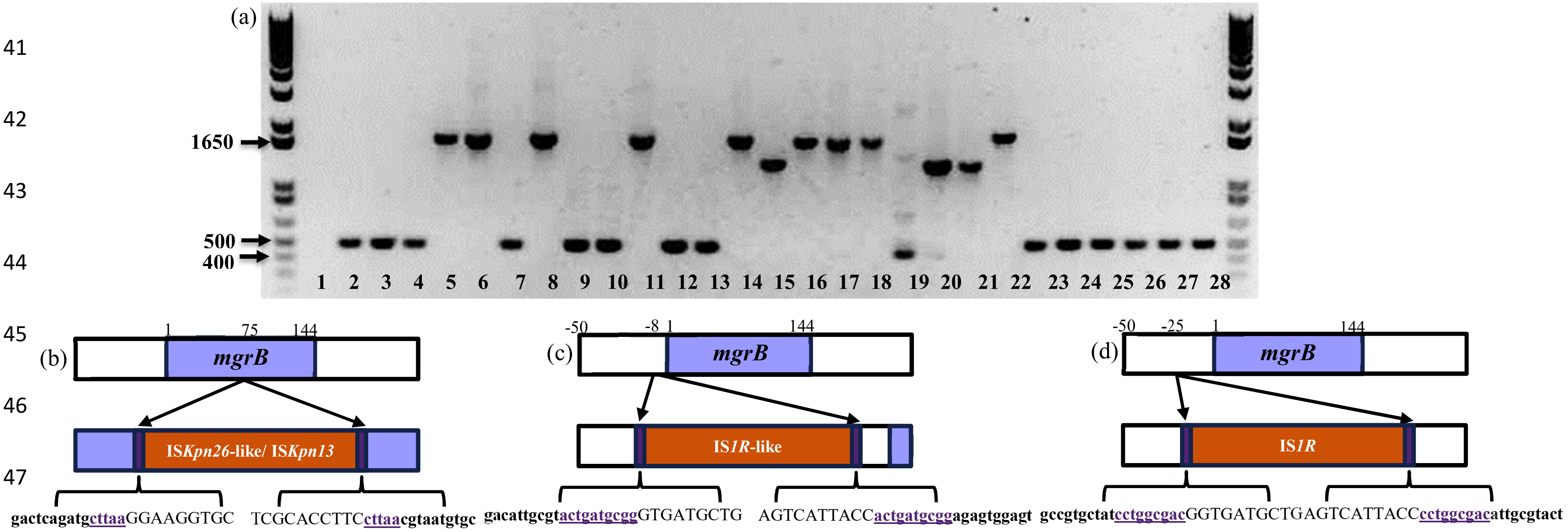

Fig. S1. Amplification of *mgrB*. (a) PCR products of 1, ATCC 25922 (*E. coli* negative control); 2, ATCC 700603; 3, ATCC 13883; 4, ATCC BAA-2146; 5, 1_GR_13; 6, 2_GR_12; 7, 3_GR_13; 8, 4_GR_12; 9, 5_GR_13; 10, 6_GR_12; 11, 7_GR_13; 12, 8_GR_13; 13, 9_GR_12; 14, 10_GR_13; 15, 11_BR_13; 16, 12_BR_13; 17, 13_GR_14; 18, 14_GR_14; 19, 15_GR_13; 20, 16_GR_13; 21, 17_GR_14; 22, 18_GR_14; 23, 19_GR_14; 24, 20_GR_12; 25, 21_GR_13; 26, 22_GR_12; 27, 23_GR_12; 28, 24_GR_13. (b) IS*5*-like (IS*Kpn26*-like or IS*Kpn13* for 12_BR_13) element producing 1700 bp amplicon. (c) IS*1R*-like element resulting in 1268 bp amplicon in 16_GR_13 and 17_GR_14 (d) IS*1R* element in 11_BR_13 resulting in 1268 bp amplicon.

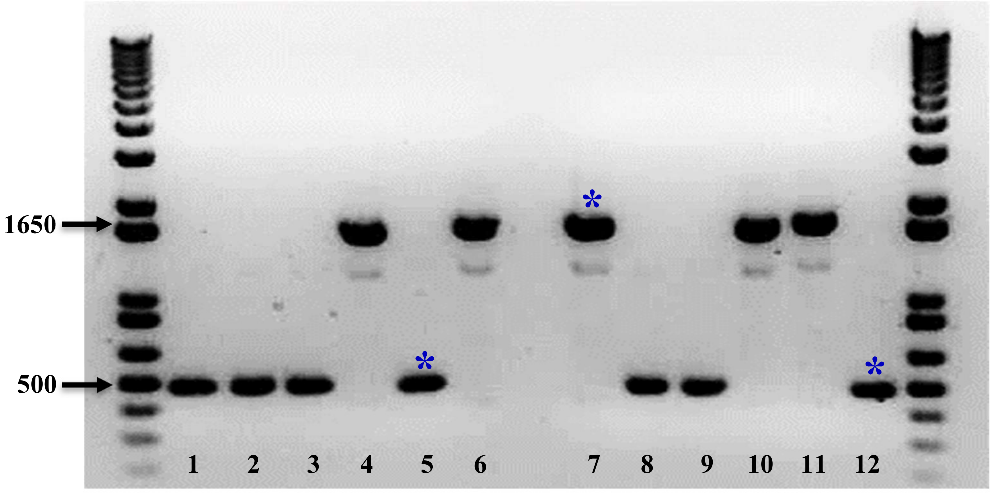

Fig. S2. Amplification of chromosomal *mgrB* for complemented 13_GR_14 (lanes 1 to 6) and 14_GR_14 (lanes 7 to 12). Complementation via pTOPO-*mgrB* is shown in lanes 1 to 3 and 7 to 9 and for pTOPO-*pmrB*, lanes 4 to 6 and 10 to 12. Three colonies were tested per treatment group and reversion to a polymyxin-susceptible phenotype is indicated by *. A 500 bp amplicon represents an intact *mgrB* whilst a 1700 bp amplicon is the result of the IS*Kpn26*-like insertion in *mgrB*.

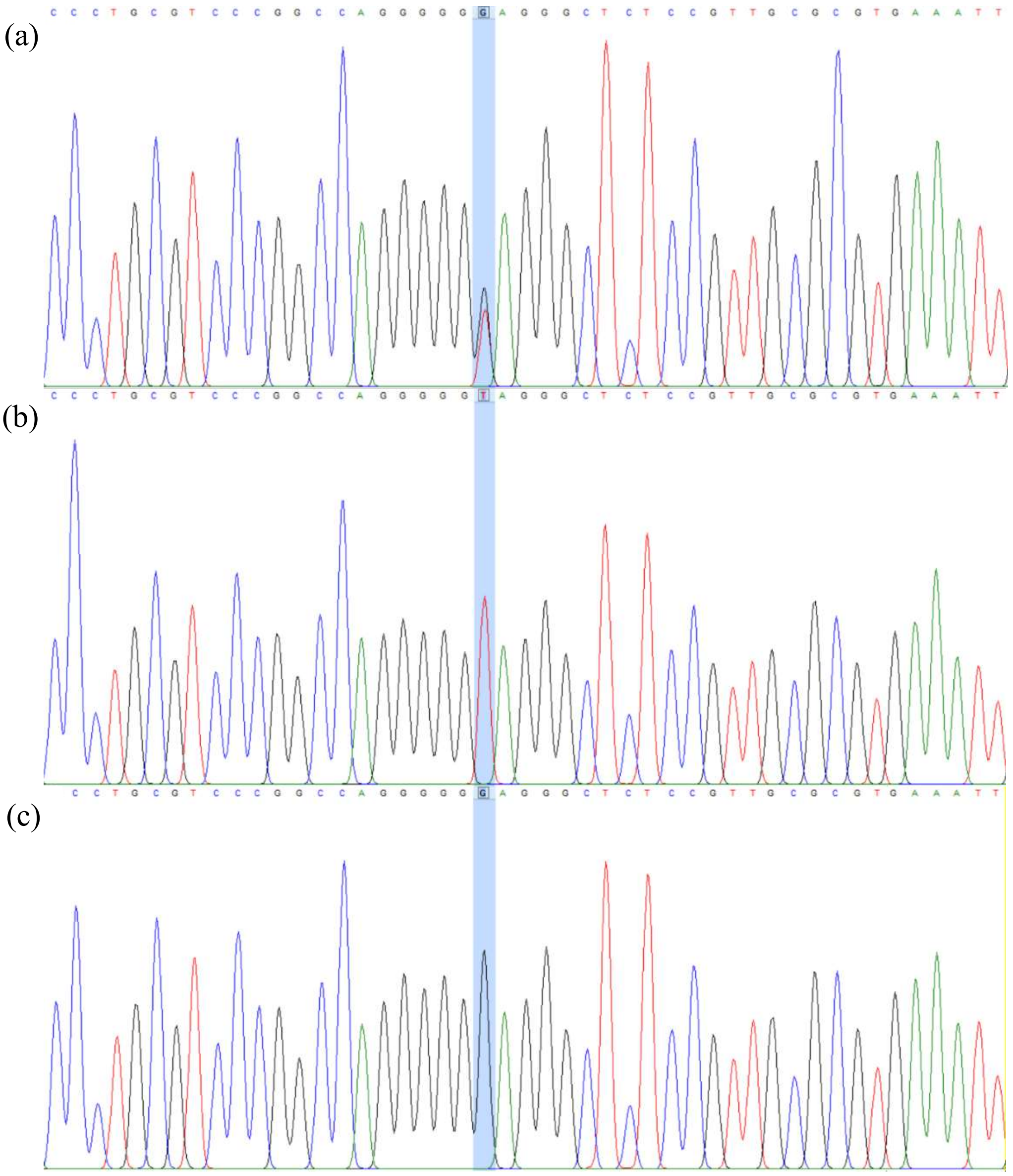

Fig. S3. Sanger sequencing traces of amplified *phoQ* from (a) DNA extraction of 14_GR_14 used for whole genome sequencing. (b) complementation of 14_GR_14 where susceptibility was recovered by pTOPO-*mgrB*. (c) complementation of 14_GR_14 where susceptibility was recovered by pTOPO-*pmrB*. WT sequence contains **T** at nucleotide position 1337 (shaded) and mutation of **G** causing V446G was isolated in condition (c).
